# An open chat with…Stuart Ferguson

**DOI:** 10.1002/2211-5463.13282

**Published:** 2021-09-21

**Authors:** Duncan Wright, Stuart J. Ferguson

**Affiliations:** ^1^ FEBS Open Bio Editorial Office Cambridge UK; ^2^ Department of Biochemistry University of Oxford Oxford UK

## Abstract

Stuart Ferguson has been on the Editorial Board of *FEBS Open Bio* since its inception, and his expertise, dedication, and support have been invaluable for the journal’s growth and success. Stuart Ferguson received his doctorate in biochemistry from the University of Oxford, before taking up a faculty position at the University of Birmingham and later returning to Oxford, where he is currently Emeritus Fellow at St Edmund Hall but still teaching and pursuing his research interests. Stuart is also a long‐term member of the Editorial Board of our fellow FEBS Press journal, *FEBS Letters*. Stuart has kindly volunteered to be the second member of our Editorial Board to be interviewed, in celebration of the journal’s upcoming 10th anniversary.

## Graphical abstract

As *FEBS Open Bio* approaches its 10th anniversary, we have decided it is an ideal time to give the members of our prestigious Editorial Board an opportunity to discuss their research, publishing, and the activities of FEBS in the virtual pages of the journal. Our second interview is with Professor Stuart Ferguson, who has been on the Editorial Board of *FEBS Open Bio* since its inception, and whose expertise, dedication, and support have been invaluable for the journal’s growth and success.

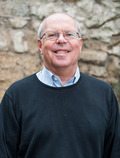

## For those who are nonspecialists, how would you describe your research?

Most of the focus in recent years has been on how cytochromes are assembled, in particular, the c‐types where the heme is covalently attached by, surprisingly, three different post‐translational sets of apparatus found distributed amongst various prokaryotes and eukaryotes. There are interesting evolutionary dimensions to this question. In addition, we have made contributions to the manner of synthesizing a specialized heme, d1 of bacterial nitrite reductase, which turned out to share steps with a previously unknown route to heme synthesis that operates in some organisms. In addition, I have had a long‐standing interest in bioenergetics in general, exemplified by the book Bioenergetics (4th edition) that I wrote with David Nicholls [1].

## What are the greatest unsolved questions in your areas of expertise?

In the very immediate area, the whole question of how heme is handled/ delivered in cells is an unknown. Given the various chemical properties of heme, it is intuitively uncomfortable to accept that it finds its own way from its site of synthesis to its end locations whether they be mitochondrial cytochromes or cytoplasmic hemoproteins. Similarly in prokaryotes, some heme has to reach the external surface of the cytoplasmic membrane. There are scarcely any clues from mutants for protein‐mediated heme transport and delivery, but negative evidence of that kind can never close the question. Elsewhere, lots of proton motive force generators, for example, Complex1 of the mitochondrial and some bacterial respiratory chains, offer difficult questions, as do many proton motive force consumers such as the Tat system for moving folded proteins, along with processes in the outer membranes of bacteria that rely on the proton motive force across the adjoining cytoplasmic membrane.

## You have been on the Editorial Board of *FEBS Open Bio* since the launch of the journal. How has the journal and the publishing landscape changed in that time?

The simplest answer is that the journal has grown fairly rapidly and at the same time has acquired, for the moment, a highly satisfactory impact factor, thus demonstrating that there is a need for a publication vehicle for the sorts of papers that *FEBS Open Bio* was set up to attract.

## What is your opinion on Open Access versus the traditional subscription publishing model?

I start with the observation that there was not one traditional publishing model. On the one hand, there are commercial publishers such as Elsevier but also Nature, Cell Press, etc., who published a whole range of journals from the very prestigious to the not so prominent and made their money by charging often high fees to libraries and in some cases individual subscribers. These attracted increasing disapproval because they were publishing work that had been funded by public bodies and/or charities who were interested in discoveries and not publications/publishers’ profits. On the other hand, there are journals published by learned societies such as the Biochemical Journal (UK) and the Journal of Biological Chemistry. Here, the income, again mainly from libraries, was used to support the scholarly activities of the sponsoring societies, but even here there was variation; some journals such as the Journal of Biological Chemistry charged their authors’ page charges, whereas others, such as the Biochemical Journal, did not. In general, those that did not impose page charges charged higher prices to libraries. A third model was that of FEBS who have always worked with commercial publishers to generate revenue for FEBS from their journals. All these journals made additional money from the sale of reprints. Most of the content of these journals was available only to subscribers, of which the majority were affiliated to institutional or company subscribers. The idea that all publicly funded knowledge should be available to all was given impetus by the move to online publication when it became feasible for an individual, almost anywhere in the world and without institutional or company connection, to gain access to all publications provided there was no paywall. I have always questioned how many such individuals exist, but to make publications truly open access, the costs of publication have had to shift to investigators and their institutions. At my own institution, the large sums allocated for this purpose were exhausted before the financial year was complete, thus leading to delays in publication. It is now understood that spending large sums of money this way instead of on research is unsustainable. The dream, of course, is deposition of papers on the internet at close to zero cost, but then who would organize review and proper presentation? Some will argue that we don’t need review*—*rubbish will sink ignored*—*but then how would we know about papers hosted only on an institutional website? Overall, I am inclined to think the traditional model had much to recommend it and it is not clear to me how the scientific community can stop profit‐driven commercial publishing, an original aim of open access, other than boycotting of certain journals.

## How has higher education changed since you began lecturing? Have these changes been for the better or worse?

Probably, the biggest change has been the use of PowerPoint in lectures and the onset of demand for lectures to be recorded. I have the feeling that this is leading to more rote learning and less acquisition of proper understanding. I have heard of students reviewing a lecture 100 times in belief that they will ‘star’ by showing that they can reproduce an entire lecture verbatim. PowerPoint lectures often rely on slides downloaded from textbooks and reviews without recognizing that different slides then have different abbreviations and conventions for topology of membranes etc. Not to mention mistakes such as showing penicillin acting in the cytoplasm of a bacterium with an active transport system to take it in there, completely the opposite of what the lecturer said! PowerPoint slides also tend to make lectures more like research presentations. Students are often left confused, although when used properly with purpose made slides, PowerPoint offers many positives.

## How have you found the experience of teaching online? Are there any advantages?

I have done a large number of one‐hour tutorial sessions. Not too bad, but I always preferred to sketch out problems on pieces of paper and get students to respond. It's more difficult and slower to do this online. But you can keep an eye on the clock effortlessly online!

## What advice would you offer PhD students today?

Before you start a PhD, take lots of advice. The apparently ‘best’ laboratory may be geographically unsuitable for you; what do you want to do afterwards? Many prospective PhD students are very naïve about job prospects for academics. In general, join an established laboratory that is on the ‘up’ and can give you an entrée to having new techniques/problems in your portfolio. The trouble with this advice is that, if followed, nobody would join a new group or work on currently unfashionable topics.

## How do you think Brexit will affect scientific research and higher education in the UK?

It will substantially reduce the number of EU staff and postdocs, which is likely to have a negative effect, although members of the general public will ask why UK citizens don’t want/are not good enough to gain such employment. It will also put an end, presumably, to the annual arrival of EU undergraduates and postgraduates owing to the fees being raised to match those charged to all other non‐UK citizens. At the undergraduate level, in my own experience, this will mainly affect students from Poland and Romania, where they produce school leavers sufficiently fluent in English to join UK undergraduate courses. Top end UK universities will lose some outstanding students, many of whom would have entered the UK employment market.

## Do you think Brexit will have any adverse effects on the involvement of researchers based at UK institutions in Horizon Europe?

Yes. I suspect that institutions within the EU will be wary and visa requirements for any work visits, however short, will prove an impediment. I suspect that many in the governing party would be happy if we had not remained associated with Horizon, reflecting the delight many have expressed that we have left the Erasmus scheme. So if the present Government wins the next election, I would not be surprised if involvement with Horizon were ended as it does not fit well with ‘Global Britain’.

## The AstraZeneca SARS‐CoV‐2 vaccine was recently developed in Oxford. Do you consider that Oxford remains a centre of innovation and discovery?

Obviously, I have to say yes. Matthew Arnold wrote many years ago that ‘Oxford is the home of lost causes and steeped in a romantic vision of a lost past.’ And it is the case that in the UK, Oxford has been seen as a humanities rather than as a science University. This is several years out of date, even if Oxford has one of the largest groups anywhere of students studying classics (Latin and ancient Greek). In recent years, John Goodenough was awarded a Nobel prize for lithium batteries, the seminal work he started while at Oxford, and Peter Radcliffe, still at Oxford, won the 2019 Nobel prize for discovering the molecular basis through which animals sense oxygen. The vaccine work is an interesting example of the value of maintaining expertise in an area that was not seen as particularly ‘hot’ pre‐COVID. So Oxford has been the location of great science (Nobel prizes for chemistry before the Second World War) for many years and I would say is ‘on the up’ compared with competitors. In some ways, this is remarkable given the much greater attention to undergraduate teaching than in many competitors in the UK and overseas.
